# Supplementary material for: Work-related stress: the impact of COVID-19 on critical care and redeployed nurses: a mixed-methods study
Source: BMJ Open. 2021 Jul 5;11(7):e051326. doi: 10.1136/bmjopen-2021-051326 (PMC8260305; doi:10.1136/bmjopen-2021-051326)
Supplement: Supplementary data [file bmjopen-2021-051326supp001.pdf]

Interview Schedule: Work-related stress: The Impact of COVID-19 on Critical Care and Redeployed Nurses: A mixed methods study

**Opening/Background Questions**	• To begin, could you tell me a little bit about you and your role?<br><br>• Were you originally trained to work in critical care, or have you been re-deployed from another part of the hospital?
**Experience of working in ICU during the pandemic**	• Can you tell me about your experience of working in critical care during the pandemic?<br><br>**Follow-up -** Could you describe how your typical working day has changed since the pandemic started?
**What are the main challenges?**	• Thinking back to your experiences of working in ICU during the pandemic can you tell me about any challenges/ the main challenges that you faced in your job?<br><br>**Follow-up -** How did that make you feel?<br><br>• Can you describe how **these challenges are different** from the kinds of challenges you would normally experience working in critical care before the pandemic?
**Job resources**	• Was there anything that helped you and your colleagues manage these daily challenges on shift?<br><br>• Thinking back, what resources were unavailable that would have been useful to help you manage daily challenges on shift?
**Exploring best and most challenging shifts**	• Can you tell me about one of your best shifts during the pandemic?<br><br>**Follow-up -** What happened that made it stand out?<br><br>• Can you tell me about one of the most challenging situations that you have encountered during the pandemic?<br><br>**Follow-up -** If you feel able, can you please tell me what happened that made it so challenging?
**Impact on well-being**	• How do you feel working during the pandemic has affected you personally in terms of your own wellbeing?<br><br>• How easy or difficult was it for you to detach from work after shift?<br><br>• Is there anything that helped you to recover in between shifts?

Transition Statement: From our first questionnaire we are aware that some staff have mentioned different wellbeing services or initiatives that were offered by their unit or the hospital throughout the pandemic. I would now like to ask you a few questions about that.

**Identifying well-being Initiatives**	• Were any "well-being initiatives/interventions" in place and available to you during the pandemic?<br><br>**Follow-up -** Can you tell me a bit more about the well-being initiative?
**Effectiveness**	• Did you find any of the well-being initiatives helpful during the pandemic?<br><br>**Follow-up -** Why do you think that was helpful/ was not helpful for you?
**Accessibility**	• In terms of the support measure you described, how easy or hard has it been for you to access this?
**Gaps in provision**	• Are there any other well-being initiatives support you would like?<br><br>• What staff support measures do you think would be helpful in future waves of the pandemic?
**Closing**	• Is there anything more that you would like to tell me that I haven't asked you
