# Supplementary material for: Work-related stress: the impact of COVID-19 on critical care and redeployed nurses: a mixed-methods study
Source: BMJ Open. 2021 Jul 5;11(7):e051326. doi: 10.1136/bmjopen-2021-051326 (PMC8260305; doi:10.1136/bmjopen-2021-051326)
Supplement: Author's manuscript [file bmjopen-2021-051326.draft_revisions.pdf]

BMJ Open

BMJ Open is committed to open peer review. As part of this commitment we make the peer review history of every article we publish publicly available.

When an article is published we post the peer reviewers' comments and the authors' responses online. We also post the versions of the paper that were used during peer review. These are the versions that the peer review comments apply to.

The versions of the paper that follow are the versions that were submitted during the peer review process. They are not the versions of record or the final published versions. They should not be cited or distributed as the published version of this manuscript.

BMJ Open is an open access journal and the full, final, typeset and author-corrected version of record of the manuscript is available on our site with no access controls, subscription charges or pay-per-view fees (http://bmjopen.bmj.com).

If you have any questions on BMJ Open's open peer review process please email info.bmjopen@bmj.com

BMJ Open

Work-related stress: The Impact of COVID-19 on Critical Care and Redeployed Nurses: A mixed methods study

Journal:	*BMJ Open*
Manuscript ID	bmjopen-2021-051326
Article Type:	Protocol
Date Submitted by the Author:	15-Mar-2021
Complete List of Authors:	Rattray, Janice; University of Aberdeen, Institute of Applied Health Sciences<br>McCallum, Louise; University of Glasgow, Nursing and Health Care<br>Hull, Alastair; University of Dundee<br>Salisbury, Lisa; Queen Margaret University Edinburgh, Division of Dietetics, Nutrition and Biological Sciences<br>Ramsay, Pam; University of Dundee, School of Health Sciences<br>Scott, Teresa; NHS Grampian, Critical Care<br>Cole, Stephen; NHS Tayside<br>Miller, Jordan; University of Aberdeen<br>Dixon, Diane; University of Aberdeen
Keywords:	COVID-19, Adult intensive & critical care < ANAESTHETICS, QUALITATIVE RESEARCH

SCHOLARONE™
Manuscripts

I, the Submitting Author has the right to grant and does grant on behalf of all authors of the Work (as defined in the below author licence), an exclusive licence and/or a non-exclusive licence for contributions from authors who are: i) UK Crown employees; ii) where BMJ has agreed a CC-BY licence shall apply, and/or iii) in accordance with the terms applicable for US Federal Government officers or employees acting as part of their official duties; on a worldwide, perpetual, irrevocable, royalty-free basis to BMJ Publishing Group Ltd ("BMJ") its licensees and where the relevant Journal is co-owned by BMJ to the co-owners of the Journal, to publish the Work in this journal and any other BMJ products and to exploit all rights, as set out in our licence.

The Submitting Author accepts and understands that any supply made under these terms is made by BMJ to the Submitting Author unless you are acting as an employee on behalf of your employer or a postgraduate student of an affiliated institution which is paying any applicable article publishing charge ("APC") for Open Access articles. Where the Submitting Author wishes to make the Work available on an Open Access basis (and intends to pay the relevant APC), the terms of reuse of such Open Access shall be governed by a Creative Commons licence – details of these licences and which Creative Commons licence will apply to this Work are set out in our licence referred to above.

Other than as permitted in any relevant BMJ Author's Self Archiving Policies, I confirm this Work has not been accepted for publication elsewhere, is not being considered for publication elsewhere and does not duplicate material already published. I confirm all authors consent to publication of this Work and authorise the granting of this licence.

For peer review only

Title Page: Work-related stress: The Impact of COVID-19 on Critical Care and Redeployed Nurses: A mixed methods study

Janice Rattray, PhD, MN, DipN, Cert. Ed. RGN, SCM. Aberdeen Health Psychology Group Institute of Applied Health Sciences, School of Medicine, Medical Sciences and Nutrition Rm114, Health Sciences Building, Foresterhill. Aberdeen AB25 2ZD
Telephone: 01224 438076 janice.rattray@abdn.ac.uk (corresponding author).

Louise McCallum MSc, BN, RN. University of Glasgow, Nursing & Health Care School. Louise.McCallum@glasgow.ac.uk

Alastair Hull MD, FRCPSYCH. NHS Tayside and Institute of Medical Sciences, University of Dundee. Alastair.Hull@nhs.scot

Pam Ramsay PhD, MSc, BSc, RN. School of Health Sciences, University of Dundee. pramsay001@dundee.ac.uk

Lisa Salisbury PhD, BSc (Hons). School of Health Sciences, Queen Margaret University. lsalisbury@qmu.ac.uk

Teresa Scott BN (Professional Development), Dip HEN (adult), NHS Grampian. Teresa.scott@nhs.scot

Stephen Cole, MBChB, BSc (Hons), FRCA, FFICM, FRCP, NHS Tayside. Stephen.cole@nhs.scot

Jordan Miller MSc, BSc. Institute of Applied Health Sciences, University of Aberdeen. jordan.miller@abdn.ac.uk

Diane Dixon PhD, B.A. (Hons), PhD, BSc (Hons), C. Psychol. Institute of Applied Health Sciences, University of Aberdeen. diane.dixon@abdn.ac.uk

For peer review only

For peer review only - http://bmjopen.bmj.com/site/about/guidelines.xhtml

Abstract:

Introduction: We need to understand the impact of COVID-19 on Critical Care (CCNs) and redeployed nurses and NHS organisations.

Methods and analysis: This is a two-phase mixed methods study (QUANT – QUAL), underpinned by a theoretical model of occupational stress, the Job-Demand Resources Model (JD-R). Participants are critical care and redeployed nurses from Scottish and three large English units.

Phase one is a cross-sectional survey replicating a pre-COVID-19 study and results will be compared with this data. Linear and logistic regression analysis will examine the relationship between antecedent, demographic, and professional variables on health impairment (burnout syndrome, posttraumatic stress symptoms), motivation (work engagement), and organisational outcomes (intention to remain in critical care nursing and quality of care). In addition, we will assess the usefulness of a range or resources provided by the NHS and professional organisations.

To allow in-depth exploration of individual experiences, phase two will be one-to-one semi-structured interviews with 25 CCNs and 10 redeployed nurses. The JD-R model will provide the initial coding framework to which the interview data will be mapped. The remaining content will be analysed inductively to identify and chart content that is not captured by the JD-R model. In this way the adequacy of the JD-R model is examined robustly and its expression in this context will be detailed.

Ethics and dissemination: Ethics approval was granted from the University of Aberdeen CERB2020101993. We plan to disseminate findings at stakeholder events, publish in peer reviewed journals and at present at national and international conferences.

248 words

For peer review only

For peer review only - http://bmjopen.bmj.com/site/about/guidelines.xhtml

Article Summary

Strengths & Limitations:

- The study has a robust theoretical framework.
- We can compare our survey results with pre-COVID-19 data.
- Our qualitative phase will provide an in-depth account of CCN and redeployed nurses’ experiences of working during the pandemic.
- We will be able to provide NHS managers with information that will provide a basis for supporting nurses during subsequent waves and future pandemics.
- Our findings will be limited to nurses only, thus excluding other healthcare workers

Key Words

Critical Care, Work-related stress, COVID-19

For peer review only

For peer review only - http://bmjopen.bmj.com/site/about/guidelines.xhtml

Work-related stress: The Impact of COVID-19 on Critical Care and Redeployed Nurses: A mixed methods study

Introduction

The contribution made by critical care nurses (CCNs) and those redeployed to critical care areas during this pandemic has been vital, and their expertise in caring for the increased number of critically ill patients, essential; thus, their well-being to allow them to continue in these roles is crucial. It has become clear during the first and now subsequent waves of COVID-19, that CCNs have been working in a highly charged environment with additional challenges such as supervising redeployed staff with limited or no intensive care experience; the high mortality rate of COVID-19 patients; delivering care using personal protective equipment; communicating with and supporting relatives at a distance, and the well-publicised potential risks to personal and family health. This may lead to an increase in work-related stress and its consequences at individual, unit, and organisational levels. Staff redeployed to intensive care units will face these issues also but have the added challenges of an unfamiliar environment and may feel they do not have the required skill set to care for these severely ill patients. In general, work-related stress in CCNs can lead to a range of physical[1] and psychological[2] sequalae that may present as 'burnout' [3,4,5.] It has been reported that the prevalence of burnout in CCNs prior to COVID-19 was around 16-33%, resulting in negative outcomes such as reduced quality of care, increase in staff sickness and increased staff turnover. Previously identified factors associated with CCN work-related stress are based on a poor-quality literature; tend to be inconsistent, with poor conceptualisation of work-related stress and a lack of an underpinning theoretical framework. Notwithstanding those concerns, it has been suggested that factors can be described as individual, job and work environment characteristics.

It is not unreasonable to assume that the COVID-19 pandemic and its intensified challenges will result in increased CCN work-related stress. In a recent study 45% of critical care staff met the threshold for at least one of severe depression and/or anxiety, PTSD or problem drinking, and nursing staff were more likely to report higher levels of distress than other staff[6]. However, Greenberg's study was unable to compare current levels of distress to those prior to the pandemic. To appreciate fully the impact of this pandemic, we need to understand how work-related stress has changed and evolved from pre- to post-COVID-19. Further, NHS boards and professional organisations have provided a range of resources to support staff (e.g. self-help guides, intensive care society well-being resources) all well-meaning, but crucially with little or no evaluation. If we are to support CCNs effectively, we need urgently to understand the impact of COVID-19, evidence the specific stressors and their importance, and how these interact and subsequently impact on CCNs and their organisations. Without this knowledge, we cannot identify confidently an appropriate range of measures to protect this vital workforce, or indeed for whom, when and how to implement them.

Our study has three central strengths. First it is theoretically informed by the Job Demand-Resource Model (JD-R)[7]. The JD-R model allows us to measure, understand and test a range of individual factors (personal resources e.g., resilience), work environment and job

For peer review only - http://bmjopen.bmj.com/site/about/guidelines.xhtml

characteristics (job demands (e.g., workload) and job resource variables (e.g., autonomy) that may lead to either negative (health impairment, reduced job satisfaction, burnout) or positive (work engagement, commitment) outcomes for staff, and importantly organisational outcomes (intention to remain and quality of care). Second, we (LMcC) have baseline data from a national study of work-related stress in CCNs conducted prior to the pandemic that used the JD-R model. This pre-pandemic data will form a baseline comparator dataset for the current study. Third, qualitative interviews will enable CCNs and those nurses redeployed to critical care to express in their own words how working during the pandemic has impacted upon them. In addition, we will evaluate the support services offered to staff over the course of the pandemic to establish whether they met staff needs when delivered and addressed the sources of stress identified by the JD-R model.

The aim of this study is to establish the: a) impact of COVID-19 on CCNs, and those nurses redeployed to critical care units, and b) prevalence of work-related stress and the perceived impact on quality of care and intention to remain in nursing. Secondary aims are to explore in detail the experiences of CCNs' and those nurses redeployed to critical care units, during the COVID-19 pandemic and to understand which service initiatives were accessed and their usefulness.

Ethics approval was granted from the University of Aberdeen CERB2020101993.

Methods

This is a two-phase mixed methods study (QUANT – QUAL). Phase One is a cross-sectional survey recruiting participants from across Scottish intensive care units, and three large English units. This phase will replicate our pre-pandemic study and use a range of validated and theoretically informed measures. Phase Two will be in-depth one-to-one interviews.

Participant Involvement: We have involved CCNs at different stages of this study. A current CCN(TS) is a co-applicant and has been involved in the design and implementation of the study; prior to finalising survey content we will 'sense check' this with several CCNS; we have a named CCN contact in each participating critical care unit; and four CCNs are members of the study steering group. The study commenced on 01.10.2020 and will complete on 30.09.2021.

Participants, Both Phases

CCNs: CCNs employed within ICUs caring for patients with level three care requirements across adult critical care units in NHS Scotland and three units in England.

Nurses redeployed to critical care areas: Those registered nurses who were redeployed to critical care areas on at least two occasions.

For both CCNs and redeployed nurses: Inclusion criteria are NMC registered nurses (RNs) with substantive part-time and full-time contracts. Exclusion criteria are unregistered staff with caring roles (auxiliary/support workers), RNs on permanent agency/bank contracts.

Sample Size

For peer review only - http://bmjopen.bmj.com/site/about/guidelines.xhtml

Survey: Power calculations have been calculated for the two mental health outcomes. A sample size of 500 (achieved in the pre-COVID-19 study with fewer units involved) will provide adequate power (80%) to detect a small effect in the GHQ-12 and the estimated prevalence of PTSD (24%) with a precision of 0.035 and confidence of 95%. Scotland has a less ethnic and racial diversity when compared to other parts of the UK. Therefore, the three additional units selected to take part in the project were identified for inclusion based, not only on their high admission rates, but also on their ability to address the need to better represent the experiences of staff from a diversity of ethnic and racial groups. The total sample eligible to participate in the pre-COVID-19 study was approximately 1224. With the inclusion of additional units, an eligible sample of approximately 2500 will be available for the current study (CNNs and redeployed nurses). The previous study achieved a recruitment rate of 48%, which if achieved here would provide a sample of n=1200.

Interviews: We will interview a purposive sample of 25 CCNs and 10 redeployed nurses. A sampling frame will ensure we recruit from a range of gender, grades, backgrounds, and ethnicity.

Measures

Demographic, Professional and Work Environment Details

Demographic variables will include age, gender, relationship status, number of children, caring responsibilities, RN band/grade, tenure on the unit, number of years nursing experience and critical care nursing experience, highest level of qualification, full/part time work and shift length.

Measures of the JD-R Model

We will replicate the validated measures used to operationalise the JD-R model in the pre-COVID-19 study. In addition, a measure of Posttraumatic Stress symptoms will be included, as this has emerged in recent literature[6]. The survey will be sense checked with six CCNs to ensure that we have not missed important variables.

Job Characteristics

Job characteristics will be measured mainly using subscales from the Questionnaire on the Experience & Evaluation of Work (QEEW 2.0)[8]

- **Job Demands** include seven QEEW 2.0 subscales (pace & amount of work: emotional load: mental load: physical effort: complexity: work organisation: role conflict) (32 items). Two profession specific sub-scales (13 items) emerged from a previous systematic review were adapted from the Customer-Related Social Stressors questionnaire (CSS)[9].
- **Job Resources** include twelve QEEW 2.0 subscales (learning opportunities: job autonomy: task clarity: feedback: relationship with supervisor: relationship with colleagues: effectiveness in achieving goals: quality: well-being focus: staffing (52 items).

- **Personal Resources** include resilience with Connor Davidson Resilience Scale (CD-RISC)[10] (10-items).

For peer review only - http://bmjopen.bmj.com/site/about/guidelines.xhtml

Outcome Measures

Health Impairment

- **Burnout Syndrome** will be measured using the 'Maslach Burnout Inventory for Health Services Survey' (MBI-HSS)[11] (22-items), a three-factor construct, capturing emotional exhaustion, depersonalisation, and personal accomplishment.
- **Posttraumatic stress symptoms** will be measured using the PCL-5[12]. This 20-item measure captures the DSM-V symptoms, consistent with the diagnosis of Post-traumatic stress disorder (PTSD) and,
- **Mental health** using the GHQ-12 (12 items)[13] a measure of mental health well-being.

Motivation

- **Work engagement** will be measured using the Utrecht Work Engagement Scale (UWES)[14] (9 items) which has a three-factor structure: vigour, dedication, and absorption.
- **Job Satisfaction** using (1-item) from the QEEW2.0[8]
- Other individual outcomes including '**Turnover'** (3-items), '**Recovery after Work'** (6-items), '**Detachment after Work'** (3-items), will be assessed using the QEEW 2.0[8].

Organisational Outcome

Intention to remain in critical care will be assessed using two subscales from the QEEW 2.0[8]: '**Certainty about future**' (3 items) & **'Changing jobs'** (3 items), **Patient Safety Culture** using a single item from the Agency for Healthcare Research and Quality Hospital Survey[15] and **Quality of care** using a 15-item questionnaire 'perceptions of care left undone[16] and a single item measure of **perceived quality**[17].

Support Services

Participants will also be asked to identify supportive resources provided by their Health Board/NHS Trust during the pandemic. They will be asked also, how often they used these resources, their accessibility, how useful they found them, and to identify any gaps in this provision.

Unit level data

We will collect unit level data to explore cross unit differences. This will include number of ICU beds, staffing levels, number and pattern of COVID-19 admissions, patient mortality rates, APACHE II scores, and details of service reconfiguration.

Phase 1: Theory based survey of occupational stress.

Recruitment

- ICU managers in each of the critical care units will be contacted to a) identify a designated unit contact/champion (this approach worked well in the initial (pre-COVID-19) study (LMcC) and b) determine the number of eligible CCNs within each unit and the number of redeployed nurses.
- The research team will contact and meet with each designated unit champion (either virtually due to the pandemic or in person) to introduce the study and clarify their role/responsibilities.

For peer review only - http://bmjopen.bmj.com/site/about/guidelines.xhtml

- The survey questionnaire will take approximately thirty minutes to complete.

Paper based responses

- Blank, sealed questionnaire packs including the survey, a participant information sheet, a form to complete for those interested in taking part in an interview and two return envelopes (survey and interview form) will be sent to unit champions to distribute to eligible participants.
- Unit champions will identify and arrange for the questionnaire packs to be distributed to CCNs and deployed nurses and identify a return area for completed packs.
- Posters describing the study will be provided to each champion around two weeks prior to data collection.
- Completed questionnaires will be placed within a sealed container on each ICU and a suitable place for redeployed staff.
- Sealed containers will be packaged by the unit champion three weeks after recruitment commences in the unit/hospital for collection by courier and delivery to the research team.

Online responses

- For those wishing to complete an online version of the survey, a link will be provided on the Patient Information Sheet (PIS) and on the paper questionnaire. In addition, a URL link and a QR code to the online version will be displayed on the study posters.

Consent

Consent will be assumed by the return of completed anonymised questionnaires. Unit identifiers only will be included to allow unit level analysis. Written consent will not be required for participants of Phase One. Participants will be provided with contact details for agencies/organisations for self-referral in the event of any emotional distress at any point during participation in this study. We will also provide participants access to a self-help psychological wellbeing toolkit (developed in one NHS Board in Scotland).

For peer review only

Phase One Analyses

Demographics of the population will be described using appropriate descriptive statistics (mean (SD), median (IQR) and number (%)). Work related stress will be compared between the pre- and post-COVID-19 cohorts using the chi squared test, t-test and ANOVA (or non-parametric equivalents). Linear and logistic regression analysis will examine the relationship between job demands, job resources, personal resources and demographics on health impairment (burnout syndrome, sleep quality, depression and anxiety), motivation (work engagement and vitality), and organisational outcomes. Structural equation modelling will be used to examine mediating, moderating and latent factors in these relationships.

Two researchers independently will map the support measures identified by staff to the constructs in the JD-R model to identify the theoretical constructs addressed by the support services offered to staff. Kappa will be used to assess agreement. By doing this the support services that target the sources of stress shown to be significant contributors to personal

For peer review only - http://bmjopen.bmj.com/site/about/guidelines.xhtml

and occupational outcomes will be identified. Importantly, this process will also identify areas of unmet need.

Phase Two: Theory based qualitative interviews with CCNs and Redeployed Nurses

To listen to and understand further CCNs experiences of working in critical care units during the pandemic, we will conduct individual interviews with staff. This will allow more in-depth exploration of the specific issues faced by CCNs in the pandemic including their strategies for dealing with the challenging environment. We plan to recruit around 25 CCNs and around 10 redeployed nurses. We will interview until data saturation is reached using an established stopping criterion[18].

We will recruit participants from units that reflect the different sizes and patient demographics. If social distancing measures are still in place, we will interview either via an on-line video platform or by phone, according to participant preference. With participants' permission interviews will be digitally voice recorded and transcribed by a University approved transcription service. We will ask participants to describe local supportive initiatives and their views on accessibility, usefulness, and effectiveness.

An initial theory-based interview guide will be developed, based on the Job Demand Resource model and the three theoretical domains known to influence individual and outcomes; job demands, job resources, and personal resources. We will use information from our pre-COVID-19 survey, prior 'sense check' interviews with CCNs, and evidence in the available literature to populate the topic areas in the interview guide; where possible (dependent upon timing), we will also use findings from our survey data. Interviews will take approximately 60mins.

Recruitment

Consenting survey participants will be selected according to a pre-determined sampling frame to ensure representation across clinical bandings and other key demographic and professional variables. Informed consent will be obtained and a suitable time for the interview arranged.

Consent

- A consent to contact form will be included in the questionnaire pack for those who wish to participate in the individual interviews.
- To ensure anonymity of their questionnaire responses, this will be returned in a separate envelope, in the return box, and will include the participant's name, banding and contact details.
- A member of the research team will contact the participant to arrange a suitable time for the interview, provide the participant with information and the opportunity to ask questions.
- The interviewer will either read each statement on the consent form to the participant (for telephone interviews) and/or share their screen in online interviews. Verbal consent will be obtained for each statement and will be recorded. Copies of the completed form will be stored securely on University servers.

For peer review only - http://bmjopen.bmj.com/site/about/guidelines.xhtml

Withdrawal Procedure: interview participants who wish to withdraw from the study can do so at any time up to the point at which the data are anonymised. Participants who withdraw during the interview will be asked for permission to retain the data already collected anonymously for analysis. Participants who wish to withdraw after the interview is complete can contact the study Research Fellow (RF) or PI.

Phase Two Analysis

Transcribed data will be analysed using both deductive and inductive processes. The JD-R model will provide the initial deductive coding framework to which the interview data will be mapped. The remaining interview content will be analysed inductively using the standard framework method[19] to identify and chart content that is not captured by the JD-R model. In this way the adequacy of the JD-R model is examined robustly and its expression in this context will be detailed. Further, the additional content can be evaluated relative to the components of the JD-R model and independently of them. By using this process, the application of theory is preserved, and our qualitative understanding builds in a cumulative theory-based manner. This method will be applied to the data that captures the personal experience of working during the pandemic and content that discusses the experience of the support services offered.

Integration of data

The application of the JD-R model to both the quantitative and qualitative data facilitates integration of the data. In this way we will be able to present a unique account of the impact of COVID-19 on this workforce. Comparisons with our pre-COVID-19 data provide an opportunity that will likely not exist elsewhere. In these unparalleled times, we do not yet know whether COVID-19 has had a sustained detrimental effect on this workforce; this is vital information to obtain.

Data Management and Data Protection

We will comply with the requirements of the General Data Protection Regulations (GDPR) and the Data Protection Act 2018. We will adhere also, where appropriate, to the current version of the NHS Scotland Code of Practice on Protecting Patient Confidentiality. Access to collated participant data will be restricted to the PI and appropriate study staff.

Study Management and Oversight

A Study Management Group consisting of the two PIs DD and JR, all Co=Investigators (Co-Is) and a dedicated RF will meet monthly and co-ordinate the study. The RF will oversee participant recruitment and conduct the individual interviews. In addition, a study steering group will include representation from social science, critical care nursing, and critical care researchers.

Discussion

There is no doubt that this is a timely and important study. However, we face a number of challenges. At time of submitting the proposal, indications were that the UK was emerging

For peer review only - http://bmjopen.bmj.com/site/about/guidelines.xhtml

from the worst of this pandemic. This has been demonstrated not to be the case and one of the challenges will be to recruit participants from an exhausted workforce.

There are a number of limitations. We are recruiting nurses only and therefore are excluding other professional groups and this approach will not give us a full picture of the COVID-19 impact on critical care staff. Our sample also will include nurses from Scotland and England only and are therefore not capturing the experiences of nurses in Wales and Northern Ireland. Although unlikely it may be that their experiences are different.

The main strengths of this study are that we have developed a robust, theoretically informed mixed methods study that we will recruit from critical care units across both Scotland and England and will represent the range of experiences from those working in small, medium, and large units. We will have questionnaire data from a large sample that will be representative of the population and in-depth interview data describing individual experiences of nurses working on the frontline during the pandemic. Importantly we will be able to compare work-related stress and its consequences in this professional group with pre-COVID data. This will provide us with unique empirical evidence of the consequences of this pandemic on the critical care and redeployed nurse workforce.

Funding

The study is funded by the National Institute for Healthcare Research 132068.

Data statement section:

Data will be stored on a secure server in the University of Aberdeen

Author contributions

JR, DD, LMcC, AH, PR, LS, TS, SC contributed to the study protocol.

JR, DD, LMcC, AH, PR, LS, TS, SC, JM contributed to the manuscript.

Word count: 3263

For peer review only

References

1. Lee S-J, Lee JH, Gillen M, KRAUSE N. Job stress and work-related musculoskeletal symptoms among intensive care unit nurses: a comparison between Job Demand-Control and Effort-Reward Imbalance Models. *Am J Ind Med* 2014;**57** (2) 214-221. .https://doi.org/10.1002/ajim.22274
2. Dollard M., Dormann C, Boyd CM, et al. Unique Aspects of Stress in Human Service Work. *Australian Psychologist* 2003 38 **(2)**: 84-91https://doi.org/10.1080/00050060310001707087
3. Zhang X, Huang D-S, Guan P. Job burnout among critical care nurses from 14 adult intensive care units in northeastern China: a cross-sectional survey. *BMJ Open* 2014; **4**; (6) http://dx.doi.org/10.1136/bmjopen-2014-004813
4. Merlani P, Verson M, Businger A, et al. Burnout in ICU caregivers: a multicentre study of factors associate to centers. *Am J Respir Crit Care Med* 2011. **184** (10): 1140-1146. https://doi.org/10.1164/rccm.201101-0068OC
5. Teixeira C., Ribeiro O, Fonseca AM, et al. Ethical decision making in intensive care units: a burnout risk factor? Results from a multicentre study conducted with physicians and nurses. *J Med Ethics* 2014. **40**(2): 97-103. http://dx.doi.org/10.1136/medethics-2012-100619
6. Greenberg N, Watson D, Hall T, et al. 2020. Mental health of staff working in intensive care during COVID-19. Occupational Medicine: doi:10.1093/occmed.kgaa220.
7. Bakker AB, Demeroti E. Job Demands–Resources Theory: Taking Stock and Looking Forward. *J Occup Health Psychol*. 2017; **22**(3): 273 https://psycnet.apa.org/doi/10.1037/ocp0000056
8. Van Veldhoven M, Prins J, Van der Laken P, et al. 2015. QEEW2.0 42 short scales for survey research on work, wellbeing and performance. SKB: Amsterdam. 2015. Available at: https://www.skb.nl/qeew
9. Dormann C, Zapf D. Customer-related social stressors and burnout. J Occup Health Psychol 2004; **9**(1): 61-82. https://psycnet.apa.org/doi/10.1037/1076-8998.9.1.61
10. Connor KM, Davidson JR. Development of a new resilience scale: the Connor-Davidson Resilience Scale (CD-RISC). *Depress Anxiety* 2003. **18**(2):76-82. https://doi.org/10.1002/da.10113
11. Maslach C, Jackson S. The measurement of experienced burnout. *Journal of Organizational Behaviour.*1981; **2**(2): 99–113. https://doi.org/10.1002/job.4030020205
12. Blevins CA, Weathers FW, Davis MT, et al. The Posttraumatic Stress Disorder Checklist for *DSM-5*(PCL-5): Development and initial psychometric evaluation. *J Trauma Stress*. 2015; **28**(6): 489-498. https://doi.org/10.1002/jts.22059
13. GHQ-12 https://www.gl-assessment.co.uk/products/general-health-questionnaire-ghq/
14. Schaufeli WB, Bakker AB, Salanova M. The measurement of work engagement with a short questionnaire: A cross-national study. Educ Psychol Meas 2006; 66(4):701-716. https://doi.org/10.1177%2F0013164405282471
15. Sorra J, Gray L, Streagle S, et al. AHRQ orra J, Gray L, Streagle S, et al. AHRQ Hospital Survey on Patient Safety Culture: User's Guide. (Prepared by Westat, under Contract No. HHSA290201300003C). AHRQ Publication No. 15-0049-EF (Replaces 04-0041). Rockville, MD: Agency for Healthcare Research and Quality. January 2016. 6. http://www.ahrq.gov/professionals/quality-patientsafety/patientsafetyculture/hospital/index.html
16. Ball JE, Murrells T, Rafferty AM, et al. Care left undone during nursing shifts: associations with workload and perceived quality of care. *BMJ Qual Saf* 2014; 23:116-125. http://dx.da.org/10.1136/bmjqs-2013-002489
17. Mallidou AA, Cummings GG, Estabrooks CA, et al. Nurse specialty subcultures and patient outcomes in acute care hospitals: A multiple-group structural equation modelling. *Int J Nurs Stud*. 2011: 48 (1);81-93. https://doi.org/10.1016/j.ijnurstu.2010.06.002
18. Francis JJ, Johnston M, Robertson C, et al. What is an adequate sample size? Operationalising data saturation for theory-based interview studies. *Psychology and Health*. 2010; **25**(10): 1229-1245. https://doi.org/10.1080/08870440903194015

For peer review only - http://bmjopen.bmj.com/site/about/guidelines.xhtml

19. Gale NK, Heath G, Cameron E, et al. Using the framework method for the analysis of qualitative data in multi-disciplinary health research. BMC Med Res Methodol. 2013. **13**(1); 1-8. https://doi.org/10.1186/1471-2288-13-117

For peer review only

For peer review only - http://bmjopen.bmj.com/site/about/guidelines.xhtml

BMJ Open

Work-related stress: The Impact of COVID-19 on Critical Care and Redeployed Nurses: A mixed methods study

Journal:	*BMJ Open*
Manuscript ID	bmjopen-2021-051326.R1
Article Type:	Protocol
Date Submitted by the Author:	09-May-2021
Complete List of Authors:	Rattray, Janice; University of Aberdeen, Institute of Applied Health Sciences<br>McCallum, Louise; University of Glasgow, Nursing and Health Care<br>Hull, Alastair; NHS Tayside<br>Ramsay, Pam; University of Dundee, School of Health Sciences<br>Salisbury, Lisa; Queen Margaret University Edinburgh, Division of Dietetics, Nutrition and Biological Sciences<br>Scott, Teresa; NHS Grampian, Critical Care<br>Cole, Stephen; NHS Tayside<br>Miller, Jordan; University of Aberdeen<br>Dixon, Diane; University of Aberdeen
**Primary Subject Heading**:	Nursing
Secondary Subject Heading:	Intensive care
Keywords:	COVID-19, Adult intensive & critical care < ANAESTHETICS, QUALITATIVE RESEARCH

SCHOLARONE™
Manuscripts

I, the Submitting Author has the right to grant and does grant on behalf of all authors of the Work (as defined in the below author licence), an exclusive licence and/or a non-exclusive licence for contributions from authors who are: i) UK Crown employees; ii) where BMJ has agreed a CC-BY licence shall apply, and/or iii) in accordance with the terms applicable for US Federal Government officers or employees acting as part of their official duties; on a worldwide, perpetual, irrevocable, royalty-free basis to BMJ Publishing Group Ltd ("BMJ") its licensees and where the relevant Journal is co-owned by BMJ to the co-owners of the Journal, to publish the Work in this journal and any other BMJ products and to exploit all rights, as set out in our licence.

The Submitting Author accepts and understands that any supply made under these terms is made by BMJ to the Submitting Author unless you are acting as an employee on behalf of your employer or a postgraduate student of an affiliated institution which is paying any applicable article publishing charge ("APC") for Open Access articles. Where the Submitting Author wishes to make the Work available on an Open Access basis (and intends to pay the relevant APC), the terms of reuse of such Open Access shall be governed by a Creative Commons licence – details of these licences and which Creative Commons licence will apply to this Work are set out in our licence referred to above.

Other than as permitted in any relevant BMJ Author's Self Archiving Policies, I confirm this Work has not been accepted for publication elsewhere, is not being considered for publication elsewhere and does not duplicate material already published. I confirm all authors consent to publication of this Work and authorise the granting of this licence.

For peer review only

Title Page: Work-related stress: The Impact of COVID-19 on Critical Care and Redeployed Nurses: A mixed methods study

Janice Rattray, PhD, MN, DipN, Cert. Ed. RGN, SCM. Institute of Applied Health Sciences, University of Aberdeen janice.rattray@abdn.ac.uk (corresponding author).

Louise McCallum MSc, BN, RN. Nursing & Health Care School. University of Glasgow, Louise.McCallum@glasgow.ac.uk

Alastair Hull MD, FRCPSYCH. NHS Tayside Alastair.Hull@nhs.scot

Pam Ramsay PhD, MSc, BSc, RN. School of Health Sciences, University of Dundee. pramsay001@dundee.ac.uk

Lisa Salisbury PhD, BSc (Hons). School of Health Sciences, Queen Margaret University. lsalisbury@qmu.ac.uk

Teresa Scott BN (Professional Development), Dip HEN (adult), NHS Grampian. Teresa.scott@nhs.scot

Stephen Cole, MBChB, BSc (Hons), FRCA, FFICM, FRCP, NHS Tayside. Stephen.cole@nhs.scot

Jordan Miller MSc, BSc. Institute of Applied Health Sciences, University of Aberdeen. jordan.miller@abdn.ac.uk

Diane Dixon PhD, B.A. (Hons), PhD, BSc (Hons), C. Psychol. Institute of Applied Health Sciences, University of Aberdeen. diane.dixon@abdn.ac.uk

For peer review only

For peer review only - http://bmjopen.bmj.com/site/about/guidelines.xhtml

Abstract:

Introduction: We need to understand the impact of COVID-19 on Critical Care (CCNs) and redeployed nurses and NHS organisations.

Methods and analysis: This is a mixed methods study (QUANT – QUAL), underpinned by a theoretical model of occupational stress, the Job-Demand Resources Model (JD-R). Participants are critical care and redeployed nurses from Scottish and three large English units.

Phase one is a cross-sectional survey in part replicating a pre-COVID-19 study and results will be compared with this data. Linear and logistic regression analysis will examine the relationship between antecedent, demographic, and professional variables on health impairment (burnout syndrome, mental health, posttraumatic stress symptoms), motivation (work engagement, commitment), and organisational outcomes (intention to remain in critical care nursing and quality of care). We will also assess the usefulness of a range of resources provided by the NHS and professional organisations.

To allow in-depth exploration of individual experiences, phase two will be one-to-one semi-structured interviews with 25 CCNs and 10 redeployed nurses. The JD-R model will provide the initial coding framework to which the interview data will be mapped. The remaining content will be analysed inductively to identify and chart content that is not captured by the model. In this way the adequacy of the JD-R model is examined robustly and its expression in this context will be detailed.

Ethics and dissemination: Ethics approval was granted from the University of Aberdeen CERB2020101993. We plan to disseminate findings at stakeholder events, publish in peer reviewed journals and at present at national and international conferences.

250 words

For peer review only

Article Summary

Strengths & Limitations:

- The study has a robust theoretical framework.
- We can compare our survey results with pre-COVID-19 data.
- Our qualitative phase will provide an in-depth account of CCN and redeployed nurses' experiences of working during the pandemic.
- We will be able to provide NHS managers with information that will provide a basis for supporting nurses during subsequent waves and future pandemics.
- Our findings will be limited to nurses only, thus excluding other healthcare workers

Key Words

Critical Care, Work-related stress, COVID-19

For peer review only

Work-related stress: The Impact of COVID-19 on Critical Care and Redeployed Nurses: A mixed methods study

Introduction

The contribution made by critical care nurses (CCNs) and those redeployed to critical care areas during this pandemic has been vital, and their expertise in caring for the increased number of critically ill patients, essential; thus, their well-being to allow them to continue in these roles is crucial. It has become clear during the first and now subsequent waves of COVID-19, that CCNs have been working in a highly charged environment with additional challenges such as: supervising redeployed staff with limited or no critical care experience; the high mortality rate of COVID-19 patients; delivering care using personal protective equipment; communicating with and supporting relatives at a distance, and the well-publicised potential risks to personal and family health. This may lead to an increase in work-related stress and its consequences at individual, unit, and organisational levels. Staff redeployed to intensive care units will face these issues also but have the added challenges of an unfamiliar environment and may feel they do not have the required skill set to care for these severely ill patients. In general, work-related stress in CCNs can lead to a range of physical[1] and psychological[2] sequalae that may present as 'burnout'. It has been reported that the prevalence of burnout in CCNs prior to COVID-19 was around 16-33%, resulting in negative outcomes such as reduced quality of care, increase in staff sickness and increased staff turnover [3,4,5]. Previously identified factors associated with CCN work-related stress are based on a poor-quality literature; tend to be inconsistent, with poor conceptualisation of work-related stress and a lack of an underpinning theoretical framework. Notwithstanding those concerns, it has been suggested that factors can be described as individual, job and work environment characteristics.

It is not unreasonable to assume that the COVID-19 pandemic and its intensified challenges will result in increased CCN work-related stress. In a recent study 45% of critical care staff met the threshold for at least one of severe depression and/or anxiety, PTSD or problem drinking, and nursing staff were more likely to report higher levels of distress than other staff[6]. However, Greenberg's study was unable to compare current levels of distress to those prior to the pandemic. To appreciate fully the impact of this pandemic, we need to understand how work-related stress has changed and evolved from pre- to post-COVID-19. It is essential to do this when staff are still able to recall experiences with some clarity but, informed by the understanding that individuals, including health care responders, experience a broad range of early reactions, including transient distress. Consistent with the principles of Psychological First Aid (PFA) we wished to allow the normalisation of these immediate responses and fostering of adaptive functioning, and instead we are interested in measuring the enduring consequences of working through the pandemic[7]. Having pre-COVID-19 data provides a comparator that can help us understand these consequences for both the individual and organisation. By using a similar cross-sectional approach and many of the same measures including burnout our pre-COVID-19 data will allow us to do this. Further, NHS boards and professional organisations have provided a range of resources to support staff (e.g. self-help guides, intensive care society well-being resources) all well-meaning, but crucially with little or no evaluation. If we are to support CCNs effectively, we need urgently to understand the

For peer review only - http://bmjopen.bmj.com/site/about/guidelines.xhtml

impact of COVID-19, evidence the specific stressors and their importance, and how these interact and subsequently impact on CCNs and their organisations. Without this knowledge, we cannot identify confidently an appropriate range of measures to protect this vital workforce, or indeed for whom, when and how to implement them.

Our study has three central strengths. First it is theoretically informed by the Job Demand-Resource Model (JD-R)[8]. The JD-R model allows us to measure, understand and test a range of individual factors (personal resources e.g., resilience), work environment and job characteristics (job demands (e.g., workload) and job resource variables (e.g., autonomy) that may lead to either negative (health impairment, reduced job satisfaction, burnout, impaired mental wellbeing) or positive (work engagement, commitment) outcomes for staff, and importantly organisational outcomes (turnover, patient safety culture, and quality of care). Second, we (LMcC) have baseline data from a national study of work-related stress in CCNs conducted prior to the pandemic that used the JD-R model. This pre-pandemic data will form a baseline comparator dataset for the current study. Third, qualitative interviews will enable CCNs and those nurses redeployed to critical care to express in their own words how working during the pandemic has impacted upon them. In addition, we will evaluate the support services offered to staff over the course of the pandemic to establish whether they met staff needs when delivered and addressed the sources of stress identified by the JD-R model.

The aim of this study is to establish the: a) impact of COVID-19 on CCNs, and those nurses redeployed to critical care units, and b) prevalence of work-related stress and the perceived impact on quality of care and intention to remain in nursing. Secondary aims are to explore in detail the experiences of CCNs' and those nurses redeployed to critical care units, during the COVID-19 pandemic and to understand which service initiatives were accessed and their perceived usefulness.

Ethics approval was granted from the University of Aberdeen CERB2020101993.

Methods

This is a two-phase mixed methods study (QUANT – QUAL). Phase One is a cross-sectional survey recruiting participants from across Scottish intensive care units, and three large English units. This phase will replicate our pre-pandemic study and use a range of validated and theoretically informed measures. Phase Two will be in-depth one-to-one interviews.

Participant Involvement: We have involved CCNs at different stages of this study. A current CCN (TS) is a co-applicant and has been involved in the design and implementation of the study; prior to finalising survey content we will 'sense check' this with several CCNs; we have a named CCN contact in each participating critical care unit; and four CCNs are members of the study steering group. The study commenced on 01.10.2020 with a planned duration of 12 months.

Participants, Both Phases

CCNs: CCNs employed within ICUs caring for patients with level three care requirements across adult critical care units in NHS Scotland and three units in England.

Nurses redeployed to critical care areas: Those registered nurses who were redeployed to critical care areas on at least two occasions.

For peer review only - http://bmjopen.bmj.com/site/about/guidelines.xhtml

For both CCNs and redeployed nurses: Inclusion criteria are NMC registered nurses (RNs) with substantive part-time and full-time contracts. Exclusion criteria are unregistered staff with caring roles (auxiliary/support workers), RNs on permanent agency/bank contracts.

Sample Size

Survey: Power calculations have been calculated for the two mental health outcomes (posttraumatic stress symptomatology and mental health). A sample size of 500 (achieved in the pre-COVID-19 study with fewer units involved) will provide adequate power (80%) to detect a small effect in the GHQ-12 and the estimated prevalence of PTSD (24%) with a precision of 0.035 and confidence of 95%. Scotland has a less ethnic and racial diversity when compared to other parts of the UK. Therefore, the three additional units selected to take part in the project were identified for inclusion based, not only on their high admission rates, but also on their ability to better represent the experiences of staff from a diversity of ethnic and racial groups. The total sample eligible to participate in the pre-COVID-19 study was approximately 1224. With the inclusion of additional units, an eligible sample of approximately 2500 will be available for the current study (CNNs and redeployed nurses). The previous study achieved a recruitment rate of 48%, which if achieved here would provide a sample of n=1200.

Interviews: We will interview a purposive sample of 25 CCNs and 10 redeployed nurses. A sampling frame will ensure we recruit from a range of gender, grades, backgrounds, and ethnicity.

Measures

Demographic, Professional and Work Environment Details

Demographic variables will include: age, gender, relationship status, number of children, caring responsibilities, RN band/grade, tenure on the unit, number of years nursing experience and critical care nursing experience, highest level of qualification, full/part time work and shift length.

Measures of the JD-R Model

We will replicate the validated measures used to operationalise the JD-R model in the pre-COVID-19 study. These will include a range of job demands, job and personal resources, health impairment, motivational, and organisational outcomes. In addition, a measure of Posttraumatic Stress symptoms will be included, as this has emerged in recent literature[6]. The survey will be sense checked with six CCNs to ensure that we have not missed important variables.

Job Characteristics

Job characteristics will be measured mainly using subscales from the Questionnaire on the Experience & Evaluation of Work (QEEW 2.0)[9] This measure consists of a number of subscales that measure different aspects of workload and job stress. It has been used internationally across a range of occupational groups and each subscale has demonstrated reliability and content validity[9]. Job demands are those aspects of a job which require physical and/or mental effort and may exert physical, psychological, and cognitive effects on an employee[10]. Job resources are aspects of a job that influence goal achievement, reduce job demands and

For peer review only - http://bmjopen.bmj.com/site/about/guidelines.xhtml

encourage personal growth and development[10]. Personal resources are characteristics that influence how well an individual deals with their work environment and how well they can control this environment[11].

- **Job Demands** include seven QEEW 2.0[9] subscales (32 items). These include: **Pace and Amount of Work** is a 6-item subscale relating to the speed and pressure of work alongside the amount. **Emotional Load** contains 5-items relating to how emotionally demanding work is. **Mental Load** contains 3-items relating to cognitive demand and precision of work. **Physical effort** includes 3-items relating to the physical demands of work. **Complexity of work** includes 3-items relating to the complexity and difficulty of work. **Work organisation** contains 6-items related to interruptions and hindrances in conducting work. **Role conflict** has 5 items related to aspects of a role that are disliked or unclear. Items included in these subscales are rated on a 4-point frequency response format from 'always' to 'never'. Two profession specific sub-scales (13 items) emerged from a previous systematic review were adapted from the Customer-Related Social Stressors questionnaire (CSS)[12]. These are 'disproportionate relative/visitor expectations' and contains 7-items capturing unrealistic demands from relatives and verbal aggression from relatives/visitors which has 6-items. Both subscales have established reliability[12].

- **Job Resources** include twelve QEEW 2.0[9] subscales (52 items) and include: **Learning Opportunities** which is a 3-item subscale related to opportunities for growth and development. **Effectiveness in achieving goals** contains 4-items related to clarity of what needs to be achieved and organisational support to meet these goals. These items are scored on a 5-point 'Likert-type' scale from 'strongly agree', to 'strongly disagree'.

  Autonomy has 4-items related to having freedom to decide or organise activities. **Task clarity** is also a 4-item subscale related to the demarcation of responsibility for specific tasks. **Feedback** contains 4-items that relate to opportunities to obtain feedback on the purpose and results of an individual's work. **Relationship with supervisor** and **relationship with colleagues** both have 6-items with the former relating to an individual's relationship with their supervisor, and the latter reflecting support and collegial nature of relations within the team. Items are rated on a 4-point frequency response format from 'always' to 'never'.

  Quality is an organisational-related job resource that includes 4-items related to the extent that quality is valued within the organisation. The final two subscales represent employment-related job resources and include **well-being focus** which has 5-items related to the extent that the organisation prioritises and values staff well-being. Both quality and well-being focus have a 'likert-type' 5-point response format from 'strongly agree' to 'strongly disagree'. Finally, **staff** which has 4-items related to the extent that staffing levels are prioritised within the organisation and has a 4-point frequency response format.

- **Personal Resources** includes resilience which is the extent to which an individual prospers in the face of hardship and reflects coping ability. The measure used to assess resilience is the 10-item Connor Davidson Resilience Scale (CD-RISC)[13]. This is a single construct scale with a 5-point response format ranging from 'not true at all' to 'true nearly all of the time'.

For peer review only - http://bmjopen.bmj.com/site/about/guidelines.xhtml

Outcome Measures

These reflect outcomes for individuals reflecting both the negative (health impairment), and positive (motivation) arms of the JD-R model and include also organisational outcomes.

Health Impairment

- **Burnout Syndrome** will be measured using the 'Maslach Burnout Inventory for Health Services Survey' (MBI-HSS)[14] (22-items), a three-factor construct, capturing emotional exhaustion (the extent to which a person feels exhausted or overwhelmed by their work), depersonalisation (captures feelings or impersonal responses towards recipients), and personal accomplishment (feelings of competence and achievement). The MBI has demonstrated reliability and construct validity[15].
- **Posttraumatic stress symptoms** will be measured using the PCL-5[16]. This 20-item measure captures the DSM-V symptoms (intrusive thoughts, avoidant behaviours, negative changes in thinking and mood and changes in physical and emotional reactions), consistent with the diagnosis of Post-traumatic stress disorder (PTSD)[17]. Participants are asked to consider how bothersome each item has been over the past month using a 4-point response format ranging from 'not at all' to 'extremely'. The PCL-5 has demonstrated reliability and validity[16] .
- **Mental health** using the GHQ-12 (12 items)[18] a measure of mental health well-being. The GHQ has one domain[19], with a 4-point response category, the wording of which differs depending on the item.

  Other individual outcomes will be assessed using the QEEW2.0[9] including **Recovery after Work** a 6-item subscale that relates to the immediate effects of work on home life, and **Detachment after Work** a 3-item subscale relating to the ability to psychologically disconnect themselves from work, hence being able to replenish resources, aid recovery and maintain health, consistent with Hobfoll's Conservation of Resources Theory (1989)[20].

Motivation

- **Work engagement** refers to a high energy, positive and fulfilling work-related state of mind and will be captured using the Utrecht Work Engagement Scale (UWES)[21] (9 items). This reliable scale[22] has a three-factor structure: vigour (high energy and resilience), dedication (sense of pride and commitment), and absorption (concentration and immersion within work).
- **Job Satisfaction** using (1-item) from the QEEW2.0[9]

Organisational Outcomes

Intention to remain in critical care will be assessed using two subscales from the QEEW 2.0[9]: Turnover is assessed using two subscales. **Certainty about future** has 3-items relating to how certain an employee is that they will be in the same position within the next year. **Changing jobs** also has 3-items and reflects the intention an individual has to keep or change their current job. Both have a 5-point 'Likert-type' response format from 'strongly agree to strongly disagree'.

Patient Safety Culture was assessed using a single item from the Agency for Healthcare Research and Quality Hospital Survey[23] that asks respondents to rate overall quality of patient safety from 'failing' to 'excellent'. **Quality of care** was assessed using a 15-item questionnaire

For peer review only - http://bmjopen.bmj.com/site/about/guidelines.xhtml

'perceptions of care left undone[24] and a single item measure of **perceived quality**[25]. **Perceptions of care undone**, asks nurses to indicate using a binary format of 'yes', 'no', against 13 activities whether care was not performed, and the number of occasions on which this occurred. The single item measure asked about in general how was the quality of nursing care from a 4-point response format ranging from 'poor' to 'excellent'. This item has performed well in previous studies involving nurses[25].

Support Services

Participants will also be asked to identify supportive resources provided by their Health Board/NHS Trust during the pandemic. They will be asked also, how often they used these resources, their accessibility, how useful they found them, and to identify any gaps in this provision.

Unit level data

We will collect unit level data to explore cross unit differences. This will include number of ICU beds, staffing levels, number and pattern of ICU admissions, both COVID-19 and non-COVID-19 , length of ICU stay, patient mortality rates, APACHE II scores, and details of service reconfiguration.

Phase 1: Theory based survey of occupational stress.

Recruitment

- ICU managers in each of the critical care units will be contacted to a) identify a designated unit contact/champion (this approach worked well in the initial (pre-COVID-19) study (LMcC) and b) determine the number of eligible CCNs within each unit and the number of redeployed nurses.
- The research team will contact and meet with each designated unit champion (either virtually due to the pandemic or in person) to introduce the study and clarify their role/responsibilities.
- The survey questionnaire will take approximately thirty minutes to complete.

Paper based responses

- Blank, sealed questionnaire packs including the survey, a participant information sheet, a form to complete for those interested in taking part in an interview and two return envelopes (survey and interview form) will be sent to unit champions to distribute to eligible participants.
- Unit champions will identify and arrange for the questionnaire packs to be distributed to CCNs and deployed nurses and identify a return area for completed packs.
- Posters describing the study will be provided to each champion around two weeks prior to data collection.
- Completed questionnaires will be placed within a sealed container on each ICU and a suitable place for redeployed staff.
- Sealed containers will be packaged by the unit champion three weeks after recruitment commences in the unit/hospital for collection by courier and delivery to the research team.

For peer review only

Online responses

- For those wishing to complete an online version of the survey, a link will be provided on the Patient Information Sheet (PIS) and on the paper questionnaire. In addition, a URL link and a QR code to the online version will be displayed on the study posters.

Consent

Consent will be assumed by the return of completed anonymised questionnaires. Unit identifiers only will be included to allow unit level analysis. Written consent will not be required for participants of Phase One. Participants will be provided with contact details for agencies/organisations for self-referral in the event of any emotional distress at any point during participation in this study. Two levels of consent will be obtained for the Phase two interviews. In the first instance, staff who volunteer to participant in the interviews will be asked to sign a 'consent to contact' slip included in the questionnaire pack. Prior to interview, written informed consent will be obtained. We will use a distress protocol developed for a recent qualitative study involving staff working in critical care during COVID-19 to guide interviewer response if a participant becomes distressed and we will also provide distressed participants access to a self-help psychological wellbeing toolkit (developed in one NHS Board in Scotland).

Phase One Analyses

Demographics of the population will be described using appropriate descriptive statistics (mean (SD), median (IQR) and number (%)). Work related stress will be compared between the pre- and post-COVID-19 cohorts using the chi squared test, t-test and ANOVA (or non-parametric equivalents). Linear and logistic regression analysis will examine the relationship between job demands, job resources, personal resources and demographics on health impairment (burnout syndrome, sleep quality, depression and anxiety), motivation (work engagement and vitality), and organisational outcomes. Structural equation modelling will be used to examine mediating, moderating and latent factors in these relationships.

Two researchers will independently map the support measures identified by staff to the constructs in the JD-R model to identify the theoretical constructs addressed by the support services offered to staff. Kappa will be used to assess agreement. By doing this the support services that target the sources of stress shown to be significant contributors to personal and occupational outcomes will be identified. Importantly, this process will also identify areas of unmet need.

Phase Two: Theory based qualitative interviews with CCNs and Redeployed Nurses

To listen to and understand further CCNs experiences of working in critical care units during the pandemic, we will conduct individual interviews with staff. This will allow more in-depth exploration of the specific issues faced by CCNs in the pandemic including their strategies for dealing with the challenging environment. We plan to recruit around 25 CCNs and around 10 redeployed nurses. We will interview until data saturation is reached using an established stopping criterion[26].

For peer review only - http://bmjopen.bmj.com/site/about/guidelines.xhtml

We will recruit participants from units that reflect the different sizes and patient demographics. If social distancing measures are still in place, we will interview either via an on-line video platform or by phone, according to participant preference. With participants' permission interviews will be digitally voice recorded and transcribed by a University approved transcription service. We will ask participants to describe local supportive initiatives and their views on accessibility, usefulness, and effectiveness.

An initial theory-based interview guide will be developed (see supplementary file), based on the Job Demand-Resource model and the three theoretical domains known to influence individual and outcomes; job demands, job resources, and personal resources. We will use information from our pre-COVID-19 survey, prior 'sense check' interviews with CCNs, and evidence in the available literature to iteratively develop the interview guide; where possible (dependent upon timing), we will also use findings from our survey data. Interviews will take approximately 60mins.

Recruitment

Consenting survey participants will be selected according to a pre-determined sampling frame to ensure representation across clinical bandings and other key demographic and professional variables. Informed consent will be obtained and a suitable time for the interview arranged.

Consent

- A consent to contact form will be included in the questionnaire pack for those who wish to participate in the individual interviews.
- To ensure anonymity of their questionnaire responses, this will be returned in a separate envelope, in the return box, and will include the participant's name, banding and contact details.
- A member of the research team will contact the participant to arrange a suitable time for the interview, provide the participant with information and the opportunity to ask questions.
- The interviewer will either read each statement on the consent form to the participant (for telephone interviews) and/or share their screen in online interviews. Verbal consent will be obtained for each statement and will be recorded. Copies of the completed form will be stored securely on University servers.

Withdrawal Procedure: interview participants who wish to withdraw from the study can do so at any time up to the point at which the data are anonymised. Participants who withdraw during the interview will be asked for permission to retain the data already collected anonymously for analysis. Participants who wish to withdraw after the interview is complete can contact the study Research Fellow (RF) or PI.

Phase Two Analysis

Transcribed data will be analysed using both deductive and inductive processes. The JD-R model will provide the initial deductive coding framework to which the interview data will be mapped. The remaining interview content will be analysed inductively using the standard framework method[27] to identify and chart content that is not captured by the JD-R model. In this way the adequacy of the JD-R model is examined robustly and its expression in this context

For peer review only - http://bmjopen.bmj.com/site/about/guidelines.xhtml

will be detailed. Further, the additional content can be evaluated relative to the components of the JD-R model and independently of them. By using this process, the application of theory is preserved, and our qualitative understanding builds in a cumulative theory-based manner. This method will be applied to the data that captures the personal experience of working during the pandemic and content that discusses the experience of the support services offered.

Integration of data

The application of the JD-R model to both the quantitative and qualitative data facilitates integration of the data. In this way we will be able to present a unique account of the impact of COVID-19 on this workforce. Comparisons with our pre-COVID-19 data provide an opportunity that will likely not exist elsewhere. In these unparalleled times, we do not yet know whether COVID-19 has had a sustained detrimental effect on this workforce; this is vital information to obtain.

Data Management and Data Protection

We will comply with the requirements of the General Data Protection Regulations (GDPR) and the Data Protection Act 2018. We will adhere also, where appropriate, to the current version of the NHS Scotland Code of Practice on Protecting Patient Confidentiality. Access to collated participant data will be restricted to the PI and appropriate study staff.

Study Management and Oversight

A Study Management Group consisting of the two PIs DD and JR, all Co=Investigators (Co-Is) and a dedicated RF will meet monthly and co-ordinate the study. The RF will oversee participant recruitment and conduct the individual interviews. In addition, a study steering group will include representation from social science, critical care nursing, and critical care researchers.

Discussion

There is no doubt that this is a timely and important study. However, we face a number of challenges. At the time of submitting the proposal, indications were that the UK was emerging from the worst of this pandemic. This has been demonstrated not to be the case and one of the challenges will be to recruit participants from an exhausted workforce.

There are a number of limitations. We are recruiting nurses only and therefore are excluding other professional groups and this approach will not give us a full picture of the COVID-19 impact on critical care staff. Our sample also will include nurses from Scotland and England only and are therefore not capturing the experiences of nurses in Wales and Northern Ireland. Although unlikely it may be that their experiences are different.

The main strengths of this study are that we have developed a robust, theoretically informed mixed methods study that will recruit participants from critical care units across both Scotland and England and will represent the range of experiences from those working in small, medium, and large units. We will have questionnaire data from a large sample that will be representative of the population and in-depth interview data describing individual experiences of nurses working on the frontline during the pandemic. Importantly we will be

For peer review only - http://bmjopen.bmj.com/site/about/guidelines.xhtml

able to compare work-related stress and its consequences in this professional group with pre-COVID data. This will provide us with unique empirical evidence of the consequences of this pandemic on the critical care and redeployed nurse workforce.

Importantly, this empirical evidence will be actionable. Using a theoretical model of stress will enable the identification of factors that predict stress at a variety of levels. Some of these factors will be amenable to intervention at the level of individual staff and others at higher levels, such as organisational factors. The individual in-depth interviews will augment the quantitative modelling to identify support resources that were helpful, those that were not as helpful and resources that were not offered but might have been helpful. Through a process of matching these resources to the sources of stress we will identify areas of met and unmet need. This actionable understanding of the sources of stress experienced during the pandemic will support more effective targeting of resources to support staff during the course of the current pandemic, should a third wave occur, and in planning for other pandemic scenarios in the future.

Funding

The study is funded by the National Institute for Healthcare Research 132068.

Data statement section:

Data will be stored on a secure server in the University of Aberdeen

Acknowledgements: we would like to acknowledge the support from our current unit contacts: Gill Arbane, Sarah Aucott, Donna Beattie, Clare Brennan, Noreen Clarke, Yvonne Dolan, Jacqueline Gaynor, Andrea Hamilton, Jennifer Howie, Catherine Jardine, Dorothy Kerr, Wendy Laurie, Corrienne McCulloch, Alison McIntosh, Lee McLeish, Louise Murphy, Pauline Murray, Pauline Ringland, Pamela Scott, Deborah Smith, David Watson,

Author contributions

JR, DD, LMcC, AH, PR, LS, TS, SC contributed to the study protocol.

JR, DD, LMcC, AH, PR, LS, TS, SC, JM contributed to the manuscript.

Competing interests: There are no competing interests.

Word count: 4458

For peer review only - http://bmjopen.bmj.com/site/about/guidelines.xhtml

For peer review only

References

1. Lee S-J, Lee JH, Gillen M, et al. Job stress and work-related musculoskeletal symptoms among intensive care unit nurses: a comparison between Job Demand-Control and Effort-Reward Imbalance Models. *Am J Ind Med* 2014; **57** (2): 214-221. https://doi.org/10.1002/ajim.22274
2. Dollard M., Dormann C, Boyd CM, et al. Unique Aspects of Stress in Human Service Work. *Australian Psychologist* 2003; 38 **(2)**: 84-91. https://doi.org/10.1080/00050060310001707087
3. Zhang X, Huang D-S, Guan P. Job burnout among critical care nurses from 14 adult intensive care units in northeastern China: a cross-sectional survey. *BMJ Open* 2014; **4**; (6) http://dx.doi.org/10.1136/bmjopen-2014-004813
4. Merlani P, Verson M, Businger A, et al. Burnout in ICU caregivers: a multicentre study of factors associate to centers. *Am J Respir Crit Care Med* 2011; **184** (10): 1140-1146. https://doi.org/10.1164/rccm.201101-0068OC
5. Teixeira C., Ribeiro O, Fonseca AM, et al. Ethical decision making in intensive care units: a burnout risk factor? Results from a multicentre study conducted with physicians and nurses. *J Med Ethics* 2014; **40**(2): 97-103. http://dx.doi.org/10.1136/medethics-2012-100619
6. Greenberg N, Watson D, Hall T, et al. Mental health of staff working in intensive care during COVID-19. *Occupational Medicine*: 2020: doi:10.1093/occmed.kgaa220
7. North Atlantic Treaty Organisation (NATO). EAPC(JMC)N(2008)0038 Psychosocial care for people affected by disasters and major incidents: a model for designing, delivering and managing psychosocial services for people involved in major incidents, conflict, disasters and terrorism. Brussels: NATO, 2008.
8. Bakker AB, Demerouti E. Job Demands–Resources Theory: Taking Stock and Looking Forward. *J Occup Health Psychol*. 2017; **22**(3): 273. https://psycnet.apa.org/doi/10.1037/ocp0000056
9. Van Veldhoven M, Prins J, Van der Laken P, et al. QEEW2.0 42 short scales for survey research on work, wellbeing and performance. SKB: Amsterdam. 2015. Available at: https://www.skb.nl/qeew
10. Demerouti E, Bakker AB, Nachreiner F, et al The Job Demands-Resources Model of Burnout. *Journal of Applied Psychology* 2001; **86**(3): 499-512. doi:10.1037//0021-9010.86.3.499
11. Schaufeli W, Taris T. A Critical Review of the Job Demands-Resources Model: Implications for Improving Work and Health. In: G.F. Bauer and O. Hämmig, Bridging Occupational, Organizational and Public Health: 43 A Transdisciplinary Approach, 2014. https://doi.org/10.1007/978-94-007-5640-3_4
12. Dormann C, Zapf D. Customer-related social stressors and burnout. *J Occup Health Psychol* 2004; **9**(1): 61-82. https://psycnet.apa.org/doi/10.1037/1076-8998.9.1.61
13. Connor KM, Davidson JR. Development of a new resilience scale: the Connor-Davidson Resilience Scale (CD-RISC). *Depress Anxiety* 2003; **18**(2):76-82. https://doi.org/10.1002/da.10113
14. Maslach C, Jackson S. The measurement of experienced burnout. *Journal of Organizational Behaviour*.1981; **2**(2): 99–113. https://doi.org/10.1002/job.4030020205
15. Loera B, Converso D, Viotti S. Evaluating the psychometric properties of the Maslach Burnout Inventory-Human Services Survey (MBI-HSS) among Italian nurses: how many factors must a researcher consider? PLoS One. 2014 Dec 12;**9**(12):e114987. doi: 10.1371/journal.pone.0114987. PMID: 25501716; PMCID: PMC4264862.
16. Blevins CA, Weathers FW, Davis MT, et al. The Posttraumatic Stress Disorder Checklist for *DSM-5*(PCL-5): Development and initial psychometric evaluation. *J Trauma Stress*. 2015; **28**(6): 489-498. https://doi.org/10.1002/jts.22059
17. Weathers FW, Litz BT, Keane TM, et al, *The PTSD Checklist for DSM-5 (PCL-5) – Standard* [Measurement instrument]. 2013 Available from www.ptsd.va.gov
18. GHQ-12 https://www.gl-assessment.co.uk/products/general-health-questionnaire-ghq/
19. Boehnke JR, Croudace TJ, Calibrating well-being, quality of life and common mental disorder items: Psychometric epidemiology in public mental health research. *British Journal of Psychiatry*, 2016; **209;** 162-168.
20. Hobfoll, S. E., 'Conservation of resources: A new attempt at conceptualizing stress', *American Psychologist* 1989; **44**:513-524.

For peer review only - http://bmjopen.bmj.com/site/about/guidelines.xhtml

21. Schaufeli WB, Bakker AB, Salanova M. The measurement of work engagement with a short questionnaire: A cross-national study. *Educ Psychol Meas* 2006; **66**(4):701-716. https://doi.org/10.1177%2F0013164405282471
22. Wickramasinghe ND, Dissanayake DS, Abeywardena GS, Validity and reliability of the Utrecht Work Engagement Scale-Student Version in Sri Lanka. *BMC Res Notes* 2018; **11;** 277. https://doi.org/10.1186/s13104-018-3388-4
23. Sorra J, Gray L, Streagle S, et al. AHRQ Hospital Survey on Patient Safety Culture: User's Guide. (Prepared by Westat, under Contract No. HHSA290201300003C). AHRQ Publication No. 15-0049-EF (Replaces 04-0041). Rockville, MD: Agency for Healthcare Research and Quality. January 2016. 6. http://www.ahrq.gov/professionals/quality-patientsafety/patientsafetyculture/hospital/index.html
24. Ball JE, Murrells T, Rafferty AM, et al. Care left undone during nursing shifts: associations with workload and perceived quality of care. *BMJ Qual Saf* 2014; **23**: 116-125. http://dx.da.org/10.1136/bmjqs-2013-002489
25. Mallidou AA, Cummings GG, Estabrooks CA, et al. Nurse specialty subcultures and patient outcomes in acute care hospitals: A multiple-group structural equation modelling. *Int J Nurs Stud*. 2011: **48** (1);81-93. https://doi.org/10.1016/j.ijnurstu.2010.06.002
26. Francis JJ, Johnston M, Robertson C, et al. What is an adequate sample size? Operationalising data saturation for theory-based interview studies. *Psychology and Health*. 2010; **25**(10): 1229-1245. https://doi.org/10.1080/08870440903194015
27. Gale NK, Heath G, Cameron E, et al. Using the framework method for the analysis of qualitative data in multi-disciplinary health research. *BMC Med Res Methodol.* 2013. **13**(1); 1-8. https://doi.org/10.1186/1471-2288-13-117

For peer review only

Interview Schedule: Work-related stress: The Impact of COVID-19 on Critical Care and Redeployed Nurses: A mixed methods study

**Opening/Background Questions**	• To begin, could you tell me a little bit about you and your role?<br>• Were you originally trained to work in critical care, or have you been re-deployed from another part of the hospital?
**Experience of working in ICU during the pandemic**	• Can you tell me about your experience of working in critical care during the pandemic?<br>**Follow-up -** Could you describe how your typical working day has changed since the pandemic started?
**What are the main challenges?**	• Thinking back to your experiences of working in ICU during the pandemic can you tell me about any challenges/ the main challenges that you faced in your job?<br>**Follow-up -** How did that make you feel?<br>• Can you describe how **these challenges are different** from the kinds of challenges you would normally experience working in critical care before the pandemic?
**Job resources**	• Was there anything that helped you and your colleagues manage these daily challenges on shift?<br>• Thinking back, what resources were unavailable that would have been useful to help you manage daily challenges on shift?
**Exploring best and most challenging shifts**	• Can you tell me about one of your best shifts during the pandemic?<br>**Follow-up -** What happened that made it stand out?<br>• Can you tell me about one of the most challenging situations that you have encountered during the pandemic?<br>**Follow-up -** If you feel able, can you please tell me what happened that made it so challenging?
**Impact on well-being**	• How do you feel working during the pandemic has affected you personally in terms of your own wellbeing?<br>• How easy or difficult was it for you to detach from work after shift?<br>• Is there anything that helped you to recover in between shifts?

For peer review only

Transition Statement: From our first questionnaire we are aware that some staff have mentioned different wellbeing services or initiatives that were offered by their unit or the hospital throughout the pandemic. I would now like to ask you a few questions about that.

**Identifying well-being Initiatives**	• Were any "well-being initiatives/interventions" in place and available to you during the pandemic?<br><br>**Follow-up -** Can you tell me a bit more about the well-being initiative?
**Effectiveness**	• Did you find any of the well-being initiatives helpful during the pandemic?<br><br>**Follow-up -** Why do you think that was helpful/ was not helpful for you?
**Accessibility**	• In terms of the support measure you described, how easy or hard has it been for you to access this?
**Gaps in provision**	• Are there any other well-being initiatives support you would like?<br><br>• What staff support measures do you think would be helpful in future waves of the pandemic?
**Closing**	• Is there anything more that you would like to tell me that I haven't asked you

For peer review only
